# Methadone Take-Home Policies and Associated Mortality: Permitting versus Non-Permitting States

**DOI:** 10.1177/29768357241272379

**Published:** 2024-08-16

**Authors:** Rebecca Arden Harris

**Affiliations:** Department of Family Medicine and Community Health, Perelman School of Medicine, University of Pennsylvania, Philadelphia, PA, USA

**Keywords:** Methadone, methadone-involved overdose deaths, take-home doses, opioid use disorder (OUD), opioid treatment program (OTP), difference in-differences, interrupted time series analysis, COVID-19

## Abstract

To mitigate COVID-19 exposure risks in methadone clinics, the Substance Abuse and Mental Health Services Administration (SAMHSA) issued a temporary modification of regulations in March 2020 to permit, with state concurrence, extended take-home methadone doses. The modification allowed for up to 28 days of take-home methadone for stable patients and 14 days for those less stable. Using both interrupted time series and difference-in-differences methods, this study examined the association between the policy change and fatal methadone overdoses, comparing states that permitted the expansion of take-home doses with states that did not. The findings suggest the pandemic emergency take-home policy did not increase methadone-involved mortality.

## Introduction

Substantial evidence supports the effectiveness of methadone treatment in alleviating withdrawal symptoms, reducing cravings, lowering the risk of fatal overdose, and improving overall functioning and quality of life in individuals with opioid use disorder (OUD).^1^ However, since 1972, when the US Food and Drug Administration approved the use of methadone for treating opioid use disorder, there has been tight governmental control through federal statutes and regulations that allow only federally certified opioid treatment programs (OTPs) to dispense the medication. Before the COVID-19 pandemic, most patients receiving methadone had to appear in person for initial evaluations and then in person every morning 5 to 6 days per week for directly observed therapy. In response to the first wave of the pandemic, the Substance Abuse and Mental Health Services Administration (SAMHSA) in March 2020 issued a blanket exception that permitted OTPs, with state concurrence, to provide up to 28 days of take-home methadone for clinically stable patients and 14 days for those who were less stable.^2^

This study examined the association between the policy change and methadone-involved overdose deaths, comparing states that relaxed take-home dose restrictions with those that did not. To our knowledge, this study is the first to analyze the association by whether the states concurred in the exception for take-home doses.

## Methods and Materials

Mortality data were obtained from the Centers for Disease Control and Prevention (CDC) on-line CDC-WONDER tools.^3^ I compiled monthly methadone-involved overdose deaths from January 2018 to June 2022 (54 months; 27 months before the federal policy change and 27 after), stratifying by whether the state permitted OTPs to extend methadone take-home doses. I used interrupted time series analysis to model trends in monthly overdose deaths.^4^ States permitting extended take-home doses were considered the treatment group (n = 42, including DC), states not permitting were the comparison group (HI, MT, WI). A state was excluded if information on methadone take-home status was inconsistent or missing (MI, SC, TX, VT), authorization was rescinded before the study period ended (OH), or the state did not have an OTP (WY).^2,5^

After testing for preintervention parallel trends, I estimated the difference in the slopes before and after the policy change (i.e. change-of-slope coefficients) for each group of states. I then compared the postintervention trends between the two groups of states, as well as the differences in their change-of-slope coefficients. Additionally, I calculated the average treatment effect on the treated (ATET) using the difference-in-differences method. A *P*-value of <.05 was considered statistically significant.

This study used publicly available, de-identified data and was deemed exempt from human subjects review.

## Results

**Figure 1.**
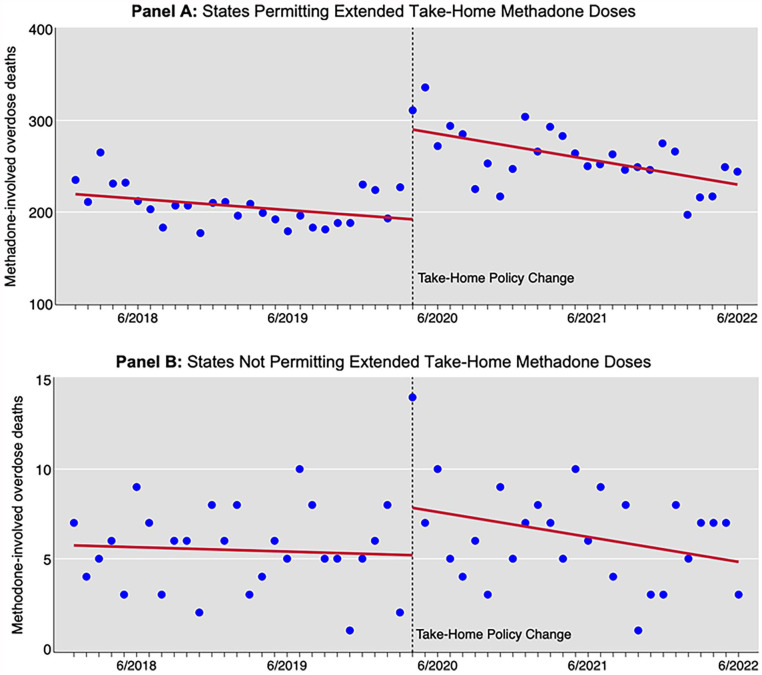
Drug overdose deaths involving methadone, January 2018 through June 2022. Circles are actual deaths, trend lines are interrupted time series estimates, and dashed vertical lines indicate start of take−home policy. Panel A: States permitting extended take-home methadone doses. Panel B: States not permitting extended take-home methadone doses. Data source: Centers for Disease Control and Prevention, National Center for Health Statistics. National Vital Statistics System, CDC WONDER Online Database.

*Preintervention Trends*: Before the policy change in March 2020, the states that subsequently permitted extended take-home doses saw a non-significant decline in methadone deaths at a rate of −1.02 per month (95% CI: −2.31, 0.28). The trend line was flat in the prohibiting states at −0.02 per month (95% CI: −0.13, 0.09). The difference between groups was −0.99 (95%CI: −2.28, 0.28), indicating the null hypothesis of parallel trends was not rejected (Figure 1).

*Change in mortality level with the onset of COVID-19*: In April 2020, both groups of states saw a sharp increase in methadone-involved deaths, estimated by subtracting the end point of the preintervention trend line (March 2020) from the start point of the postintervention trend line. In states permitting extended take-home doses, the increase was 98.02 deaths (95% CI: 65.32, 130.72), a 50.8% increase from March 2020. In states not permitting the change, the increase was 2.64 deaths (95% CI: −0.50, 5.78), a 50.6% increase.

*Postintervention trends*: Following the policy change, states permitting extended take-home doses saw the rate of deaths decrease at −2.31 per month (95% CI: −3.86, −0.76). In states not extending take-home doses, there was a nonsignificant decrease in the rate of deaths post-intervention at −0.12 per month (95% CI: −0.26, 0.03). The difference between the 2 groups was significant at −2.19 deaths per month (95% CI: −3.73, −0.66), with states that permitted extended take-home doses showing greater reductions in monthly mortality.

*Average treatment effect on the treated*: The ATET estimate was 52.89 additional deaths per month (95% CI: 38.61, 67.17) in the states allowing extended doses.

*Pre-post change in slope*: In states permitting extended doses, the rate of decrease before and after the policy change changed by −1.29 (95% CI: −3.36, 0.78), and in non-permitting states, by −0.10 (95% CI: −0.28, 0.09).

*Difference-in-differences in slope*: Between the 2 groups, the difference in the change of slope before and after the policy change was not statistically significant at −1.19 (95% CI: −3.24, 0.85).

## Discussion and Conclusions

This study suggests the policy change allowing extended take-home doses did not result in an increase in monthly methadone-involved overdose deaths in states that adopted the practice. The trend lines in both groups of states were generally similar, with the exception that states allowing extended take-home doses had larger decreases in mortality rates after the intervention. The abrupt increase in the level of methadone mortality that began in April 2020, and which occurred in both groups, was likely due to disruptions related to COVID-19. Similar increases in mortality level occurred in drug overdose deaths not involving methadone and in related domains such as alcohol-associated deaths.^6,7^ The ATET estimate predominantly captured the impact of pandemic disruptions as the statistic is more sensitive to level shifts than underlying trends.^8^

A criticism directed at the federal policy change is that easing restrictions might increase the risks of overdose and diversion.^9,10^ This study did not find any evidence of a link between extended take-home doses and increased mortality. Moreover, recent research has shown that the extended take-home policy was associated with fewer methadone-related deaths among Black and Hispanic men.^11^ A possible explanation for the decrease is the demeaning experience of having to daily report to an OTP for Black and Hispanic men, who are already marginalized and continually exposed to systems of surveillance, stigma, and punishment. The provision of additional take-home doses introduced a semblance of normalcy and dignity that was absent with regular OTP attendance. This change reduced how often patients needed to visit clinics and gave them more control over their treatment, potentially enhancing adherence and overall well-being. The extended take-home policy may have lowered certain social and economic barriers to treatment, like transportation costs and conflicts with work schedules, which disproportionately affect marginalized communities. Also, this added flexibility could have strengthened the therapeutic alliance between healthcare providers and patients by fostering trust and increasing patients’ involvement in their treatment.

Similarly, available research suggests that diversion of methadone was low among patients receiving extended take-home doses.^10^ Nevertheless, even with the strict protocols governing methadone treatment, informal sharing sometimes occurs, often to help friends or family members manage the pain of opioid withdrawal.^12^ The impact of such diversion on the illicit drug market appears minimal, given methadone’s limited psychotropic effects.^13,14^

This study has limitations. First, the non-permitting group of states was small, which precluded the ability to control for potential confounders. Second, this study could not determine whether the individuals who died from methadone-involved overdoses received the methadone through OTPs (about 90% of all methadone supplies in the US are distributed to OTPs), or from pharmacy dispensed prescriptions for pain (about 9%), or from other sources, including diverted methadone.^15^ Of note, Kaufman et al.’s cross-state analysis of methadone supplies found no association between the distribution of methadone for pain and methadone overdoses, reducing concerns about misuse of methadone pain prescriptions.^16^ Third, approximately 5% of death certificates did not list the drugs involved in the overdose.^17^

In February 2024, SAMHSA updated federal methadone regulations to permanently expand take-home dosing flexibilities, although states and local OTPs can still choose whether to participate.^11^ In a contrasting development, some states, including Arizona, Florida, Indiana, Ohio, and Mississippi, have rescinded their policy of permitting extended take-home privileges.^5^ New York state also has seen a trend toward more restrictive dosing.^18^ These policy decisions significantly influence patient experience, treatment protocols, and health outcomes.^19^ Continued or reinstated restrictions on methadone dosing may impede patients’ medication adherence and their ability to meet treatment goals. Future research should explore factors influencing state and OTP methadone dose restrictions, their treatment impacts, and opportunities for patient-centered policy development. This includes investigating political, economic, and social influences on decisions, and assessing long-term patient outcomes. Researchers should also identify potential disparities in implementation across demographic groups and regions. This research will contribute to advancing evidence-based guidelines for improving OUD treatment.

## Supplemental Material

sj-docx-1-sat-10.1177_29768357241272379 – Supplemental material for Methadone Take-Home Policies and Associated Mortality: Permitting versus Non-Permitting States Supplemental material, sj-docx-1-sat-10.1177_29768357241272379 for Methadone Take-Home Policies and Associated Mortality: Permitting versus Non-Permitting States by Rebecca Arden Harris in Substance Abuse: Research and Treatment
