# Supplementary material for: Methadone Take-Home Policies and Associated Mortality: Permitting versus Non-Permitting States
Source: Subst Use. 2024 Aug 16;18:29768357241272379. doi: 10.1177/29768357241272379 (PMC11331457; doi:10.1177/29768357241272379)
Supplement: sj-docx-1-sat-10.1177_29768357241272379 – Supplemental material for Methadone Take-Home Policies and Associated Mortality: Permitting versus Non-Permitting States [file sj-docx-1-sat-10.1177_29768357241272379.docx]

**Methadone Take-Home Policies and Associated Mortality:**

**Permitting vs. Non-Permitting States**

Rebecca Arden Harris, MD MSc

**Supplement**

**Interrupted time series analysis (ITSA) regression model**

The standard interrupted time series regression model takes the form:

*Y_t_* = *B*_0_ + *B*_1_*T_t_* + *B*_2_*X_t_*  + *B*_3_*X_t_T_t_* + e*_t_*

where

*Y_t_* is the outcome variable measured at each time point *_t_*

*T_t_* is the time since the start of the study

*X_t_* is a dummy variable representing the intervention (pre-intervention periods 0, otherwise 1)

*X_t_T_t_* is an interaction term

*B*_0_ is the intercept

*B*_1_ is the slope prior to the intervention

*B*_2_ is the change in the level of the outcome variable immediately following the introduction of the intervention (compared to the counterfactual)

*B*_3_ is the difference between the pre- and post-intervention slopes of the outcome, and

e*_t_* is the error term.

Interrupted time series analysis (single-group *ITSA* in Stata) was used to assess whether there was a change in the level or trend in monthly methadone-involved deaths following the SAMHSA take-home policy change. The pre-intervention period was January 2018 to March 2020. The post-intervention time period was April 2020 to June 2022. The model constructs a counterfactual – what the trend would look like in the absence of the policy change – which is then compared with the post-intervention actual trend.

Multiple-group *ITSA* was used to estimate the difference in post-intervention trends and the difference in change of slope coefficients between the two groups of states (states that permitted and states that prohibited extended take-home methadone doses).
